# Clinical Report on Cases Observed at the New Orleans Charity Hospital

**Published:** 1859-08

**Authors:** Austin Flint

**Affiliations:** Prof. of Clin. Med., &c., in the New Orleans School of Medicine


### NEW YORK MONTHLY REVIEW,

AND

### BUFFALO MEDICAL JOURNAL.

**VOL. 15.** AUGUST, 1859. **NO. 3.**

### ORIGINAL COMMUNICATIONS.

ART. I.—*Clinical Report on cases observed at the New Orleans Charity Hospital·* By AUSTIN FLINT, M. D., Prof. of Clin. Med. etc., in the New Orleans School of Medicine.

### DISEASES OF THE HEART.

SEVERAL interesting and instructive cases of disease of the heart were received in my wards during the session of 1858–9; and through the kindness of my brethren of the medical staff of the Hospital, I had an opportunity of observing cases which occurred in other wards. I shall select for this report, the cases which illustrate important points relating to the diagnosis, pathology, and treatment of cardiac diseases; and after giving, as succinctly as possible, an account of each case, I shall notice briefly, under the head of *Remarks*, the particular points in the clinical history, which claim the attention of the reader. In no province of practical medicine have the evidences of progress, during the last quarter of a century, been more marked than in that which embraces the diseases of the heart. The discrimination of these diseases, by means of physical exploration, may now be made with great precision; and the advancement in pathological and therapeutical views, although not so easily defined as improvements in diagnosis, is not less positive. But, it must be confessed, the attention bestowed by medical students and practitioners in general, on the study of this class of diseases, is not commensurate with their frequency of occurrence, their importance, and the facility with which the study may be prosecuted. It is, perhaps, not doing injustice to the medical profession of this country to say, that a large proportion do not undertake to make themselves practically conversant with the means of determining the existence of cardiac disease, and of recognizing the various lesions to which the heart is liable. More than this, not a few profess to be distrustful of the progress made, within late years, in diagnosis, especially as regards the reliability of physical signs, and in this way attempt to reconcile themselves to their own neglect of the subject, while they disparage the knowledge and skill which, by dint of industry, others have acquired. Reports of cases are highly useful if they serve in any measure to correct these false notions; and in inviting attention to the few cases which will form the basis of this paper, I may use the language of an esteemed author, which I have borrowed on a previous occasion :* "Happy am I in my own estimation if I have stimulated the zeal of our young practitioners for the diagnostic studies which constitute, in my mind, one of the most beautiful parts of our art."

CASE 1. *Aortic valvular lesions. Moderate enlargement of the heart. Coexisting anœmia. The anœmia relieved by medical treatment, and the patient discharged complaining of no symptoms referable to the heart.*

Dan Mullen, aged 18, Irish, laborer, admitted Dec. 4, 1858. He stated that he had been unable to work for the preceding four months, and that he had not been perfectly well for several years. His chief difficulties for the four months before his admission, related to the action of the heart and respiration. He was subject to palpitation and want of breath on exercise. Two years before, he had intermittent fever; and for eighteen months he had frequent relapses, but no relapse had occurred during the last four months. He had never had rheumatism, nor any acute chest affection.

His aspect denoted marked anæmia. The prolabia appeared bloodless. He was able to be up, dressed and out of doors. He had been in the hospital twice previously, the first time four, and the second time two years before, and, as he stated, for the same ailments as now. Appetite was good, and no inconvenience felt from taking food. The bowels were constipated. No cough. Suffered from pain in the head and giddiness. He was easily put

---

* Prize Essay, 1852. From Andry on Diseases of the Heart.

out of breath on exercise. The pulse was 80, and small, but regular in rhythm. Respirations 20. Urine acid and not albuminous. No œdema.

*Physical Signs.*—A feeble apex-beat was felt in the fifth intercostal space on the vertical line of the nipple; and a stronger, synchronous impulse in the fourth intercostal space, just within the nipple. Areas of deep and superficial dullness on percussion, were somewhat greater than normal. The first sound of the heart at the apex was normal, with slight tinnitus; the valvular element was sufficiently marked. No heaving of the præcordia. A systolic murmur, slightly rough, was heard, with greatest intensity in the second intercostal space on the left side; feeble over the body of the heart, and still more so over the apex. It was propagated into the carotids. The second sound of the heart was more intense on the left than on the right side of the sternum at the base; and it was apparently aortic on the left, as well as on the right side. Strongly marked venous hum existed on the right side of the neck, instantly and invariably suspended by deep pressure at the upper part of the neck.

The treatment consisted of the citrate of iron and quinia, with full diet.

Dec. 20th. The treatment having been continued steadily without variation, the anæmia had nearly disappeared, and he reported himself quite well, complaining of no symptoms referable to the heart. He was discharged at his own request, feeling able to go to work. The venous hum had disappeared. The other physical signs remained without any change.

*Remarks.*—This case presents a combination occurring not unfrequently, and highly important in a practical point of view, viz., organic disease of the heart and anæmia. In such a case there is a two-fold liability to error. The practitioner may attribute all the symptoms and the signs to the anæmia, especially if the latter be strongly marked. The palpitation, dyspnœa on exertion, etc., would admit of this explanation. The venous hum proceeds from this condition. And the cardiac murmur might be considered as inorganic or anæmic. A circumstance favoring the latter conclusion in the case just reported, was the murmur having its greatest intensity on the left side of the sternum at the base of the heart—a circumstance pointing to the pulmonary artery as the source of the murmur. With this view of the case, the cardiac affection would be regarded as purely functional. This would have been an error. The cardiac murmur was due to valvular lesions. This was shown by its roughness of quality, and its persistence after the anæmia had in a great measure disappeared, the venous hum having ceased. Moreover, the heart was clearly although moderately enlarged, as shown by the increased areas of superficial and deep dullness on percussion, the existence of impulse in two intercostal spaces, and the removal of the apex-beat to the left as far as the vertical line of the nipple. Although the cardiac murmur had its maximum of intensity on the left side of the sternum (which is an exception to the rule), it emanated from the aorta. This was shown by its being propagated into the carotids. The fact, too, of the second sound of the heart having the same characters, as regards quality and pitch, on the left as on the right side of the sternum, as well as greater intensity, showed that, for some reason, in this case the aortic sound was transmitted more readily to the left than the right second intercostal space; the reverse obtaining in the majority of cases. This exception may have been owing to a slight change in the position of the heart, altering the normal relations of the aorta and pulmonary artery to the intercostal spaces.

On the other hand, the existence of organic disease having been determined, the practitioner may overlook the effects of the anæmia, and attribute all the symptoms to the organic disease. This error is more unfavorable to the interests of patients than the other. The organic disease was not sufficient, in the case reported, alone to give rise to much, if any inconvenience. The palpitation and dyspnœa were, in fact, due to the anæmia, or at least to the combination of anæmia and organic disease. When the anæmia was removed, these and other symptoms disappeared, and the patient reported himself quite well. In this point of view, the case is highly instructive. It happens not unfrequently, that a patient presents this combination of affections, when the distressing symptoms proceed mainly from the abnormal condition of the blood. The practitioner is liable to attribute all the symptoms to the cardiac lesions; and he consequently errs as regards the prognosis and treatment. I have met repeatedly with cases in which patients appeared to be in an advanced stage of organic disease of the heart, anasarca, even, being added to other symptoms, when, after removing the causes inducing anæmia (for example, lactation), and resorting to proper measures of treatment, the cardiac disease, so far as symptoms are concerned, seemed to disappear. Physical exploration, however, showed the continuance of signs denoting certain lesions; but, without the co-existing anæmia, these remained innocuous.

It is not always easy to determine at once, when organic disease and anæmia are combined, to what extent the symptoms are referable to the anæmia. This becomes apparent after a time, when the anæmia disappears under the use of chalybeate tonics, stimulants, nutritious diet, and out-door exercise.

But the uncertainty, at first, as regards this point, does not affect the treatment. The therapeutical indications relate to the anæmia. It is always a prime object to remove this condition if possible. It may be laid down as a rule of practice, to which there are hardly any exceptions, whenever organic disease of the heart is associated with anæmia, the latter is to take precedence as regards treatment, and any measures addressed to the former, conflicting with those which are indicated by the anæmic condition, will do harm rather than good.

CASE 2. *Aortic valvular lesions, with regurgitation. Mitral valvular lesions, with regurgitation. Considerable enlargement of heart. Absence of symptoms denoting cardiac disease.*

Hugh Horan, aged 11, admitted Dec. 5th, 1858. He stated that he was ill for a week only, prior to his admission, having previously been perfectly well. He is an intelligent lad, replying to questions clearly, and he declared that he has never been troubled with palpitation or want of breath; that he had been able to engage in sports as actively as other boys, and had never felt any inconvenience from violent exercise. He had never had rheumatism, nor any acute disease of the chest that he was aware of. Pulse 92, small and weak. Respirations 40.

*Physical Signs.* The præcordia was abnormally prominent. The apex beat was felt in the fifth intercostal space, and extended half an inch without the nipple. The area of impulse in this interspace extended an inch. An impulse synchronous with that in the fifth, was also seen and felt in the fourth intercostal space, directly below the nipple. The fifth rib was raised with each ventricular systole. An artery of considerable size was seen and felt crossing the eighth rib, two or three inches to the left of the xyphoid cartilage. A soft systolic murmur had its maximum of intensity just without the apex, and was heard behind, near the lower angle of the scapula. It was heard over the body, and feebly at the base of the heart. At the base, on the right side of the sternum, in the second intercostal space, there was a rough systolic murmur. In the second and third intercostal spaces, near the sternum, on both sides, a systolic and diastolic murmur were present. The diastolic murmur was not heard elsewhere. The rough systolic murmur at the base, was heard with considerable intensity behind, at the upper part of the intercostal space. There were distinctly two murmurs behind; one low in pitch and rough at the upper part of the interscapular space, gradually diminishing as the stethoscope is carried downward; the other acute and soft, having its maximum behind, near the angle of the scapula, was diffused over the left lateral surface of the chest, and was loudest near the apex of the heart, preserving in these situations, its characters as regards softness and pitch. The rough systolic murmur emanating from the base of the heart, was propagated into the carotids. The second sound of the heart was notably distinct and loud. This sound was louder in the right than in the left second intercostal space, and no disparity as regards the pitch or quality of this sound existed between the two sides. The valvular element of the first sound over and near the apex-beat, was wanting; an element of impulsion was alone heard, and this was not marked.

The treatment in this case consisted of the sulphate of quinia, grs. ij, three times daily, and full diet.

On Dec. 8th the patient reported much better. The pulse was 64 and the respirations 20.

Dec. 11th, he reported quite well; and, desiring to leave the hospital, he was discharged. The physical signs remained the same as at the time of his admission.

*Remarks.* This patient entered for a febrile attack (ephemeral fever) which apparently had no connection with the disease of the heart. After remaining in hospital six days, the febrile movement had disappeared, and he declared that he was perfectly well, as he had been up to a few days before his admission into hospital. Yet there existed in this case aortic lesions, permitting regurgitation, as shown by the systolic and diastolic murmurs referable to the aorta, together with mitral regurgitant lesions, as shown by the systolic murmur referable to the mitral orifice. Moreover, the heart was considerably enlarged, as shown by the situation of the apex-beat, the existence of two impulses, together with increased extent and degree of dullness on percussion. Hypertrophy predominated over dilatation, as shown by the force of the impulses, and the elevation of the fifth rib with each systole. The mitral lesions were more serious than the aortic, as shown by the extinction of the valvular element of the first sound of the heart, whilst the aortic second sound was scarcely if at all impaired.

The practitioner but little acquainted, practically, with diseases of the heart, might naturally be led to suppose that with aortic and mitral lesions, and also considerable enlargement, the condition of the patient must involve inconvenience, if not imminent danger. Yet this patient, a lad of eleven years, was able to participate fully in the active sports of childhood, and had never been conscious of palpitation or dyspnœa. Not a few instances of this description have fallen under my observation. Organic disease of the heart, especially in early life, as regards symptoms, is often entirely latent. Patients not only do not present symptoms pointing to the heart as the seat of disease, but they may appear to be in perfect health. The cardiac affection is often discovered, as it were, accidentally; physical explorations being resorted to without any expectation of finding any abnormal signs referable to the heart. The immunity from distress or inconvenience, may continue for many years. Sooner or later, however, if the patient be not carried off by some intercurrent affection, the symptoms of organic disease are declared.

The innocuousness, for an indefinite period, of lesions giving rise to valvular insufficiency and great enlargement, is a fact of great practical importance, not only in its bearing on prognosis, but as regards the pathology and management of organic diseases of the heart. In the great majority of cases, cardiac lesions have been innocuous for a long time before coming under the cognizance of the physician. In adults, they date often from an attack of rheumatism which occurred five, ten, fifteen or twenty years before. Now, what renders these lesions latent for such a length of time? In answer to this question, it may be said, the lesions, progressively increasing, and involving, more and more, either obstruction or regurgitation, or both, finally lead to so much disturbance of the circulation that the results are manifested. This explanation is not complete. As a rule, valvular lesions are well borne, and often remain latent, so long as their immediate effects on the circulation through the heart are compensated for by hypertrophy of the organ. Hypertrophy is a conservative provision to obviate evils which would otherwise flow from valvular lesions. The natural tendency of obstructive or regurgitant lesions, is to induce hypertrophy. This occurs usually prior to dilatation; and so long as hypertrophy exists without much dilatation, the patient is comparatively safe, and perhaps even free from annoyance. But hypertrophy has its limit. Sooner or later it ceases to be progressive, and dilatation becomes predominant. Then the symptoms of cardiac disease are manifested; and from this epoch the condition of the patient involves suffering and danger. What is the practical inference from these facts? Plainly, that hypertrophy is to be encouraged rather than repressed. The treatment designed to lessen the hypertrophy occurring as a complication of valvular lesions, is, in fact, hurtful in proportion as it is successful. Dilatation and feebleness of the heart are the results to be dreaded and, if possible, postponed. Hence, with the practical views which have heretofore prevailed, it has been fortunate for patients when the existence of cardiac disease has not been discovered; since, if the discovery were made, they were subjected to treatment addressed to the hypertrophy.

Hence, patients with valvular lesion live longer, and with more comfort, if the body is well supported by nutritious food and habits of tolerably active exercise are maintained, than when they are placed on reduced diet, and the utmost quietude enjoined. In short, the rule of practice in cases of valvular lesions, is to nurture the vigor of the system, and the heart's strength, so as to postpone, as long as possible, the evil day when dilatation predominates over hypertrophy.

CASE 3. *Mitral Lesions. Enlargement of the heart. Pulmonary emphysema. The cardiac disease latent.*

John Neil, aged 31, Irish, laborer, admitted December 10th, 1858.

He stated that twelve years ago he had acute articular rheumatism. He was confined to the bed for twelve days, and was not aware that he had any chest affection. He was well, and able to work constantly after the attack of rheumatism until two years before his admission, when, as he thought, he took a severe cold and was confined to the bed for a month. Prior to this there had been no palpitation nor want of breath on exercise.

A dry cough continued after the attack just mentioned, and after a couple of months he began to expectorate; cough and expectoration continued ever afterward. Both were slight, and had remained stationary, except at times when they were increased by a fresh attack of bronchitis, He had continued to work steadily up to three weeks before coming to the hospital. His cough then became more troublesome, the expectoration more abundant, and he experienced want of breath on exercise. He had had similar attacks repeatedly before, but less severe. The dyspnœa led him to enter the hospital.

On examination of the chest, the physical signs of emphysema of the left lung were sufficiently distinct, viz., vesiculo-tympanitic sonorousness over the left side of the chest; the left infra and supra clavicular regions abnormally full; absence of superior costal respiratory movements, on forced breathing, on the left side, while they were sufficiently marked on the right side; the respiratory murmur, vesicular and intense on the right side, feeble on the left side, and frequently accompanied by dry râles on the latter side.

The apex-beat was feebly felt in the fifth intercostal space, about one-eighth inch within the nipple. Deep dullness on percussion, or, in other words, the left border of the heart, extended nearly to the nipple. A soft systolic murmur was heard, having its maximum of intensity just without the nipple. It was diffused over the left lateral surface of the chest, and heard behind, at the lower angle of the scapula. It was quite indistinct over the body of the heart, and was lost at the base. The first and second sounds were notably more distinct and loud, at the base, on the *right* than on the left side.

The treatment in this case consisted of the iodide of potassium, gr. v., three times daily, a little morphia, and full diet. This treatment was continued steadily until January 18th, 1859. The chlorate of potassa was then substituted for the iodide of potassium. Progressive improvement, as regards cough, expectoration, and want of breath on exercise, continued up to February 4th, 1859, when the patient felt sufficiently well to leave the hospital. The cough and expectoration, at the time of his discharge, were quite small. The patient was confident of his ability to return to labor, The physical signs at the time of his discharge were essentially the same as when he was admitted.

*Remarks.*—The mitral valvular lesions, in this case, probably originated in the attack of rheumatism twelve years before the patient came under my observation. These lesions involved considerable regurgitation and moderate enlargement of the heart. The regurgitation was shown by the diffusion over the left lateral and posterior surface of the chest, of the mitral regurgitant murmur. Yet, the cardiac affection had been latent, and except for the coexisting pulmonary emphysema, would have remained innocuous. In fact, after the bronchitis with which the emphysema was associated had been relieved, the patient complained of no symptoms referable to the heart. But in the course of time cardiac symptoms will become developed; and, eventually, the patient will suffer from the evils of organic disease of heart and emphysema combined, and both affections progressively increasing.

It will be noticed in the account of the physical signs, that the second sound of the heart, at the base, was more distinct and loud on the right, than on the left side of the sternum. The reverse is usually observed where mitral regurgitation has led to cardiac enlargement, owing to the diminished current of blood through the aorta and the reinforcement of the pulmonic sound, in consequence of hypertrophy of the right ventricle. The coexistence of emphysema of the left lung affords an explanation of the exception to the rule in this case. The pulmonic second sound may have been, in reality, more intense than the aortic; but, owing to the interposition of emphysematous lung, the former was not transmitted to the ear of the auscultator so readily as the latter.

The combination of organic disease of heart and pulmonary emphysema, is not very infrequent. In this case the previous history shows that the former preceded the latter. The importance of exploring the whole chest, after cardiac disease has been discovered by the physical signs, is a point which this case may serve to enforce; for, otherwise, the emphysema being overlooked, all the symptoms would have been referred to the heart, while, in fact, they were due mainly to the emphysema. Incidentally, the efficacy of the iodide of potassium and the chlorate of potassa in relieving the bronchitis, which is frequently associated with emphysema and serves to perpetuate the latter, may be referred to. These remedies often appear to exert a therapeutical effect, under these circumstances, which is truly remarkable.

CASE 4. *Pulmonic and tricuspid lesions. Slight aortic lesions. One fold of the pulmonic valve wanting. Death. Autopsy.*

John Morris, aged about 30, admitted Dec 15th, 1858.

This patient entered with indefinite ailments, and the previous history was not ascertained. The case did not receive much examination on the first day, and, the patient reporting and appearing much better on the two succeeding days, a full examination was deferred. The patient made no complaint of symptoms pointing to the heart as the seat of disease; but my attention was called to the existence of an endocardial murmur by one of my private pupils, Dr. Alston, of Texas. This murmur was systolic, emanating from the base of the heart, and notably more marked in the second intercostal space on the *left*, than on the *right* side of the sternum. A feeble murmur was heard over the carotids. The aortic second sound, as compared with the pulmonic, had its normal relative intensity. It is not noted whether auscultation was practiced over the lower border of the heart.

On the fourth day after his admission, the patient presented, at my morning visit, great prostration, constant jactitation, and spasmodic inspiration. He shortly became comatose, and died during the forenoon.

The autopsy was limited to the chest and abdomen. The results did not explain the occurrence of sudden coma and death; and the case possesses interest only from a comparison of the cardiac lesions with the physical signs. The latter were not observed as fully as they would have been had the condition of the patient appeared to indicate immediate danger.

The heart, on examination after death, was found to be moderately enlarged. The walls of the left ventricle, at their thickest part, were estimated to be $\frac{3}{4}$ of an inch in thickness. The cavity was not enlarged. The right ventricle was neither hypertrophied nor dilated. The aortic valve presented on one of the semilunar folds a warty vegetation of the size of a pea, near the free extremity and situated on the ventricular aspect of the fold. The fold itself was thickened and projected into the artery. The lesions occasioned slight obstruction, but probably not regurgitation. The aortic orifice was neither contracted nor dilated. The mitral valve was perfectly normal. The pulmonic valve consisted of but two semilunar folds, a rudimentary fold being situated between the two. Both folds were considerably thickened by deposit of lymph or fibrin on their ventricular portion. The folds were not contracted, and the valve was probably sufficient. The pulmonic orifice was slightly contracted. The tricuspid valve presented on the auricular surface a considerable mass of solidified fibrin, attached pretty firmly to the anterior segment. The mass was nearly as large as a small filbert. The segments were not contracted; the orifice was neither contracted nor dilated, and the valve probably was sufficient. The right auricle was not enlarged. It was greatly distended with liquid blood, and contained a few small, black, loo-e coagula. The autopsy was made three hours after death. The foramen valve was open sufficiently to admit a goose-quill.

The liver and spleen were greatly enlarged, the latter being four or five times its average size, and the former extending into the left hypochondrium.

*Remarks.* Tricuspid and pulmonic lesions are extremely rare; but, in addition to this case, in the two cases which will follow, lesions were situated in the right side of the heart. In the foregoing case, the contraction of the pulmonic orifice and the absence of one of the folds of the semilunar valve, were congenital; and Dr. Peacock's researches show that in connection with malformations which are usually seated in the right side of the heart, consecutive valvular lesions are frequently observed.* The murmur heard with its maximum of intensity in the second intercostal space on the left side of the sternum, doubtless emanated from the pulmonic orifice. An aortic murmur had existed, which was propagated into the carotids.

The lesions were innocuous. There are no grounds for concluding that they were concerned in the illness and death of the patient.

---

* On Malformations, etc. of the Heart. London, 1858.

CASE 5. *Tricuspid lesions. Undulatory movements, ventricular and auricular, of the jugular veins. Autopsy.*

This patient was not in my ward; and my notes of the case consist mainly of the results of physical examinations made by myself, together with the condition of the heart found after death as communicated to me by Dr. Mercer, Dr. Alston, and Mr. Devon, members of my private class. At the time of my examinations there was great distension of the abdomen from ascites, and the feet were enormously œdematous. The respirations were frequent and labored. The patient was unable to lie down, and suffered extremely from dyspnœa.

*Physical Signs.* A rough systolic murmur existed at and a little below the base of the heart, and was wanting over the apex. It was most marked on the left side of the sternum. It was propagated into the carotids. The veins of the neck were enormously distended, and presented a varicose appearance. Undulations in the external jugular were observed, occurring independently of respiration. These undulations did not correspond with the arterial pulse, one occurring before, and the other nearly synchronously with, the pulsation of the radial artery. This comparison was repeated several times with the same result. The venous pulsations were seen, but not felt. They were much more distinct on the right than on the left side, but the venous distension was more marked on the left side. When the circulation in the external jugular was arrested by pressure with the finger, the vein remained full, and it filled from below when emptied by pressure upward with the finger. Other points relating to physical signs were not noted.

The patient died in a few days after my examinations. The account of the morbid appearances of the heart, as furnished by the gentlemen who have been named, showed great obstruction at the right auriculo-ventricular orifice, from the presence of a firm false membrane, producing, with the exception of two small holes admitting a goose-quill, occlusion of this orifice. The right auricle was greatly enlarged, and its walls thickened. Both ventricles were hypertrophied and the cavities dilated. The pulmonary, aortic, and mitral valves, were free from lesions.

*Remarks.* The interesting point in this case is the double undulation observed in the jugular vein, taken in connection with the extreme obstruction at the tricuspid orifice. The first of these undulatory movements, or that which preceded the radial pulse, must have been due to the contraction of the right auricle; the second undulation, or that occurring nearly synchronously with the radial pulse, probably proceeded from the systole of the right ventricle. I am not aware that the attention of clinical observers has been directed to the occurrence of a double venous pulsation, *i. e.* two pulses for each revolution or beat of the heart.

The appearances of the heart after death do not afford a clear explanation of the systolic murmur heard at the base of the heart with its maximum of intensity on the left side of the sternum.

The obstruction at the tricuspid orifice sufficiently accounts for the venous congestion, and general dropsy, symptoms which in this case were extremely marked.

CASE 5. *Large accumulation of calcareous deposit within the right ventricle. Tricuspid obstruction. Obliteration of pulmonic valves. Occlusion of the right branch of the pulmonic artery by an embolus detached from a calcareous mass within the right ventricle. Fatty degeneration, and attenuation, of the walls of the right ventricle. Pulmonary tuberculosis.*

A heart presenting the extraordinary lesions enumerated in the above caption, was taken from a subject in the dissecting-rooms of the New Orleans School of Medicine, and presented to me by Dr. Groll, Demonstrator of Anatomy in the School of Medicine.

I saw the patient on one occasion at the Charity Hospital, through the courtesy of Dr. Nichols, Demonstrator of Anatomy in the University of Louisiana, and made a cursory examination of the chest. He presented the signs and symptoms of advanced pulmonary tuberculosis. A feeble, endocardial, systolic murmur was heard at the apex of the heart, and not at the base. The stethoscope was not carried to the lower border of the heart. A murmur, as Dr. Nichols informed me, had existed at the base, and was observed to diminish and finally disappear as the heart became weakened.

The heart was considerable enlarged in volume, the transverse diameter exceeding the vertical. The right ventricle exceeded the left in length and width. The normal relations of the two ventricles, as regards size, were reversed. The weight of the organ, including the branch of the pulmonary artery containing the calcareous mass, weighed 13½ oz. The right auricle was greatly dilated, and the left auricle moderately so. The mitral and aortic valves were normal. The right ventricle was traversed by a column of calcareous matter, an inch in length and about the size of the thumb. It was attached to the muscular band connected with the anterior curtain of the tricuspid valve near the ventricular septum, and extended across the ventricle to the anterior wall. The anterior curtain was pressed downward so as nearly to close the tricuspid orifice, producing extreme obstruction at this orifice. The other curtains could not be seen. Rough, projecting masses of calcareous deposit were attached just below the situation of the pulmonic valve, but not extending to the site of this valve. The folds of this valve appeared to have shrunken away; their vestiges only existed. In the left branch of the pulmonic artery there was a mass of calcareous matter as large as a pullet's egg, which occluded entirely this artery. This calcareous mass evidently had been detached from another mass within the right ventricle, situated just below the site of the pulmonic valve; the surface from which the former had been broken off from the latter was apparent. The mass within the artery was, thus, an embolus derived from the right ventricle. The right ventricle contained an abundance of soft, dark coagula and colorless fibrin. The open spaces between the trabeculæ were filled with soft, gelatinous-like fibrin. At the lower extremity, the walls of this ventricle were quite thin—not more than two lines in thickness. Everywhere the walls of this ventricle were attenuated. The ventricular walls, especially of the right ventricle, presented, in a marked degree, the gross and microscopical appearances of fatty degeneration. The organ was extremely soft and flabby.

The tuberculous disease of the lungs was most advanced on the right side, *i. e.* the side in which the pulmonic current was unobstructed, the left branch of the pulmonic artery being entirely occluded.

*Remarks.* I had an opportunity only of examining the appearances which the heart presented after death. It would have been interesting to have ascertained whether the smaller branches of the pulmonic artery contained deposits of calcareous matter. The interest of the case consists in the remarkable character and extent of the lesions, and their situation in the right side of the heart. The connection of these with the physical signs, is not satisfactory; but my examination of the patient was brief, and made a day or two before death, when the action of the heart was extremely feeble.

Case 6. *Aortic lesions. Enlargement. Angina pectoris. Death. Autopsy.*

George Snow, aged about 40, laborer, applied for medical aid at the Dispensary of the New Orleans School of Medicine, Dec. 30, 1858, and was referred to me by my colleague, Prof. Crawcour. His ailments had commenced two months before. Prior to that date he had never felt any want of breath, or palpitation, even, on the most violent exertions. He had been accustomed to extremely hard labor, in loading boats, etc. He had always considered himself as a robust man, up to the preceding two months. Had never had rheumatism.

His ailments distinctly denoted angina pectoris. He suffered from severe pain, in paroxysms, referred to the præcordia and left forearm, not shooting into the shoulder, but limited to the forearm, and accompanied by a sense of paralysis of the muscles. The heart palpitated violently during these paroxysms, as if, to use his own words, "it would jump out of the mouth." The paroxysms at first were slight. They gradually became more intense, and also more frequent, and had obliged him to give up work a month before he applied at the Dispensary. At the time of his application, they recurred daily, and were excited by any active exertion. A paroxysm was induced by the effort of mounting the stairs at the college. The paroxysms lasted usually about five minutes. When perfectly quiet, in the intervals of the paroxysms, he was free from pain, and the action of the heart was tranquil. The paroxysms were sometimes excited by the act of eating. Were it not for these paroxysms, he felt sure that he would be able to work without difficulty. His muscular power was good; his aspect healthy; his appetite and digestion excellent, and he complained of nothing but the paroxysms.

*Physical Signs.*—Moderate heaving of the præcordia was felt on palpation. The apex-beat was in the sixth intercostal space, slightly without the vertical line of the nipple. An impulse was also felt in the fifth intercostal space, just within the nipple. In both situations the impulse was strong and sluggish. The extent and degree of dullness on percussion in the præcordia, were considerably greater than normal. The outer limit of the deep cardiac region was without the nipple. In the right second intercostal space, near the sternum, a faint, soft murmur, accompanied the systole. It was heard only in that situation. Over the body of the heart, near the left margin of the sternum, a murmur accompanied the diastole. This murmur was feeble, soft, and low in pitch. No other murmur was discovered. The second sound of the heart was distinct in the second intercostal space on the right side, but louder on the left side. This sound was lost at the left nipple and apex. At the nipple, the first sound alone was heard, and was purely valvular, the element of impulsion being eliminated when the stethoscope was carried to this point. Over the apex, the element of impulsion predominated strongly over the valvular element.

The patient was not again seen by me till Feb. 3, 1859, when he was admitted into my ward at the Charity Hospital. In the mean time, the paroxysms of angina had become more and more frequent and severe. They now occurred at short intervals, and were excited by slight causes, such as a little muscular effort, and the act of eating solid food. His sleep was disturbed by dreams which appeared to him to produce paroxysms, and he was afraid to sleep from an indefinite apprehension. He had not been able to lie down for many nights before his admission to the hospital. The paroxysms were accompanied by dyspnœa, and a sense of suffocation.* In the intervals, he breathed without difficulty. There was considerable œdema of the lower extremities. The pulse was regular, and had considerable volume. It was not notably jerking, and the visible pulsations of the superficial arteries was not marked.

A diastolic murmur (aortic regurgitant) only, was observed after his admission into hospital. Death occurred suddenly, during a paroxysm, on the second day after his admission.

On examination after death, there were no evidences of pericarditis; but the surface of the heart presented numerous small patches of ecchymosis. The heart weighed 18 oz. The transverse and longitudinal diameters were equal, viz., $6\frac{3}{8}$ inches. The form of the organ was not altered. The enlargement was due mainly to the increased size of the left ventricle. The walls of the left ventricle were 3-4 of an inch in thickness, and the cavity dilated. The walls of the right ventricle were 1-8 of an inch in thickness, and the cavity not dilated. Auricles of equal capacity, as determined by the eye. Mitral and tricuspid orifices presented nothing abnormal. The walls of both ventricles presented the appearance of healthy muscular tissues; no change denoting fatty degeneration. The deposit of fat was rather more abundant than usual on the right ventricle near the base. The pulmonic valves were perfectly normal. The aortic valves were also healthy—no deposit or thickening, or alteration of size. Above the valves, the aorta presented notable disease. The artery was dilated. At the base of the semilunar folds the dilatation was not marked, the circumference measuring $3\frac{5}{8}$ inches. An inch above the upper margins of the folds, the circumference was $4\frac{}{4}$ inches. The dilatation extended as far as the artery was removed with the heart, viz., from two to three inches. The coronary arteries were not obstructed. There was no calcification, and a probe penetrated freely for a considerable distance. The lining membrane of the aorta above the valve was opaque, velvety, easily stripped off. The fibrous coat was thick and firm; no atheroma anywhere. On both lateral

---

* It is stated incorrectly by Watson (Lectures), that dyspnœa does not enter into paroxysms of angina.

surfaces of the artery just above the upper margins of the valvular folds, were cavities precisely equal in size and general aspect. They presented the appearance of perforations, as large as a half-dime, leading, on each side, to a *cul de sac* half an inch in depth. These cavities were filled with coagula, firm but not adherent. About an inch above these cavities on the right side, were two irregular depressions, each of the size of a dime, resembling ulcers with smooth edges, the fibrous coat apparently eroded. An inch above, on the left side of the artery, was a pouch-like dilatation of the size of a filbert, the fibrous coat wanting.

*Remarks.*—Angina pectoris is an affection always dependent on organic disease of the heart; but the particular morbid condition with which it is connected, is yet to be ascertained. It is a very rare affection, while organic disease of the heart is sufficiently common. It occurs in connection with different forms of organic disease; but there is doubtless some undiscovered feature common to these different forms, which stands in immediate relation to the phenomena of angina. The morbid appearances of the heart in the foregoing case have been given with minuteness of detail, in view of our ignorance of the proximate cause of angina. The affection in this case was associated with disease of the aorta and enlargement of the heart. Statistics show that it is oftener connected with lesions situated at or near the aortic orifice, than elsewhere; and perhaps this is the sum of our present knowledge concerning its source. The appearances in this case disprove two hypotheses which have been offered on the subject, one of which attributes the angina to calcification of the coronary arteries, and the other to fatty degeneration of the heart. The latter hypothesis is maintained by some distinguished writers. In a negative point of view, *i. e.*, as disproving these hypotheses, the case is of value.

It is worthy of remark that in this case, although the aortic disease and the enlargement of the heart must have existed for some time prior to the occurrence of the angina, there had previously been no symptoms pointing to cardiac or any disease. The patient was able to take violent exercise without inconvenience; and, apparently, as he supposed, if the paroxysms of angina could have been averted, he would not have been compelled to give up labor.

CASE 7. *Rupture of the heart. Fatty degeneration.*

A subject received from the hospital, for dissection, at the New Orleans School of Medicine, presented, on opening the chest, the pericardial sac largely distended with blood. All that could be learned of the history of the case was, the patient had been admitted with delirium tremens, and died suddenly without any suspicion having been entertained of the cause of death. The subject was a male, and the appearance denoted about sixty years of age.

The heart was enlarged. Its weight was a little over 14 oz. The walls of the left ventricle were not thickened, not exceeding half an inch. The walls of the right ventricle were attenuated. The cavities were dilated. The external surface of the heart presented opaque patches, giving to it a mottled appearance. The greater portion of the walls of the right ventricle appeared to consist of fat, the muscular tissue being reduced to a thin layer, not more than a line in thickness. Examined with the microscope, by Dr. Grall, fat globules were found in great abundance; but he did not succeed in displaying the muscular fibres, the specimens becoming dried before the examination was made.

A rent was situated at the upper part of the right ventricle sufficiently large to admit readily the forefinger. A layer of fibres at the inner surface of the ventricle was ruptured, and detached over a space larger than the perforation through the ventricle. The rupture was on the anterior surface of the ventricle, near the pulmonic orifice.

CASE 8. *Pericardial friction-sound simulated by cardiac pleural friction-sound. Pleuritis. Pulmonary tuberculosis. Death. Autopsy.*

James Walsh, aged 35, laborer, admitted January 12th, 1859. He stated that he had yellow fever in 1847, and a year ago, acute rheumatism. With these exceptions, he had always been well, until three months ago, when he began to cough, and had continued to cough and expectorate moderately ever since; but he did not consider himself really ill until after an injury which he received on the chest by falling on the curbstone, eighteen days before his admission. Pain in the chest and difficulty of breathing followed this accident, and obliged him to keep the bed for two days. He was afterward up and about, but the pain in the chest continued; and being again obliged to take to the bed, he came to the hospital. On his admission he complained of pains on coughing, and taking a deep inspiration, referred to the præcordia, and the lower, posterior portion of the chest on the left side. The pulse was 132, regular, but small. Respirations 16.

*Physical signs.*—Flatness on percussion at the base of the left side, extending, behind, upward an inch above the lower angle of the scapula.

Some variation of the limit of the flatness on comparison in the sitting and semi-recumbent postures. The resonance in front, over the upper third of the chest, on the left side, notably greater than on the right side, and vesiculo-tympanitic in quality. Respiratory murmur relatively feeble at the upper portion of the chest on the left side. Absence of respiratory murmur on the left side below the line of flatness on percussion; vocal fremitus and resonance also wanting.

No apex-beat or impulse to be felt anywhere. A well-marked cardiac friction-sound, most marked within and above the level of the nipple, was heard over a space three or four inches in diameter. It was not propagated beyond the heart, except for a short distance to the left of the nipple. The sound varied in intensity with different movements of the heart. It was sometimes heard with the systole and diastole, and sometimes with the systole only. It was somewhat rough. It was intensified by firm pressure with the stethoscope. The sound was heard when respiration was suspended. It was most marked at the end of an inspiration.

This friction-sound persisted for several days, and was listened to by a great number of persons, as an excellent specimen of a pericardial friction-sound.

Delirium tremens became developed. The patient was violently delirious, requiring to be removed from the ward. Death occurred on the ninth day after his admission.

On examination after death, the left pleural sac was found to contain a large quantity of turbid serum. The left lung was pressed upward over the heart, so as to abolish the superficial cardiac region. It presented a wrinkled appearance, and crepitated slightly on pressure. The pleural surfaces were united by tender adhesions. The lung extended downward, in front, to the level of the nipple. The pericardial sac contained a moderate quantity of transparent serum. A single, small, flocculent portion of lymph only was observed at the bottom of the liquid. The pericardial surfaces were smooth and polished, presenting no trace of inflammation. The valves of the heart were perfectly normal.

The pleural surface over the entire left lung was opaque, rough, and presented a granular appearance, but no masses of lymph.

The lungs were tuberculous. At the summit of the right lung a cavity existed as large as a common walnut, surrounded by pulmonary tissue solidified by crude tubercle. The lower lobe was filled with small, crude tubercles. The left lung contained, at the interior portion of the upper lobe, a tuberculous mass of the size of a common walnut, the central portion being semi-liquified.

*Remarks.*—This case does not properly belong in the category of diseases of the heart, but the propriety of its introduction in this connection is sufficiently obvious. The cardiac friction-sound was considered as evidence of pericarditis coexisting with the pleuritis. In my clinical remarks at the bedside, the occurrence of a cardiac pleural friction-sound was stated; but the intensity, persistance, and situation of the sound, in this instance, led me to refer it to the pericardial surfaces. The situation of the compressed lung, as determined after death, accounted for the production of the sound, which was produced either by attrition between the surfaces of the pericardium and pleura in apposition, or between the pleural surfaces over the heart.

Walshe gives, as the discriminating circumstances relating to a cardiac pleural friction-sound, 1st—fixedness at one or more points; 2d—suspension of the sound by holding the breath at the end of a full inspiration; 3d—unsteadiness, irregularity, and occasional loss of the friction-sound. These three tests were not sufficiently employed;—but the first was not available, since the sound was heard over the greater part of the præcordia; the second was probably not applicable in this instance, for the sound was most intense at the end of an inspiration; and the third, although true in this case, is certainly also true in certain cases of pericarditis. In a similar case, with the advantage of the experience derived from this instance, I am unable to see how the non-existence of pericarditis could be positively determined.

